# Isolated Bilateral Adrenal Metastasis in Castration-Resistant Prostate Cancer: A Case Report and Literature Review

**DOI:** 10.7759/cureus.75985

**Published:** 2024-12-18

**Authors:** Deniz Barali, Abdullah Gul, Birol Ocak, Mehmet Ozer

**Affiliations:** 1 Urology, Bursa Yuksek Ihtisas Training and Research Hospital, Bursa, TUR; 2 Medical Oncology, Bursa Yuksek Ihtisas Training and Research Hospital, Bursa, TUR; 3 Pathology, Bursa Yuksek Ihtisas Training and Research Hospital, Bursa, TUR

**Keywords:** adrenal gland metastasis, bilateral adrenalectomy, castration-resistant prostate cancer, metastasectomy, prostate cancer progression

## Abstract

Prostate cancer frequently metastasizes to regional lymph nodes and bones, but metastasis to the adrenal glands remains rare, particularly in isolation. This case report presents an unusual instance of bilateral adrenal metastasis in a patient with castration-resistant prostate cancer. This case emphasizes the clinical relevance of adrenal metastasis in castration-resistant prostate cancer, highlighting the potential role of aggressive treatment strategies such as metastasectomy in isolated adrenal involvement, aiming to contribute to the limited literature on this rare metastatic pattern. Further studies are warranted to explore the therapeutic impact of adrenalectomy in similar cases, potentially enhancing progression-free survival in selected patients.

## Introduction

Prostate cancer ranks as the second most commonly diagnosed malignancy in men and is the sixth leading cause of cancer-related mortality worldwide [1]. Metastasis from prostate cancer, aside from regional lymph nodes and bone involvement, occurs in approximately 15-20% of cases [2]. Notably, metastases to the kidneys or adrenal glands are observed in only 1% of prostate cancer patients, with solitary metastases to these organs accounting for merely 0.3% of such cases [3]. This article aims to elucidate the progression of bilateral adrenal gland metastases in the context of a patient with castration-resistant metastatic prostate cancer, supported by a review of the current literature.

## Case presentation

**Figure 1:**
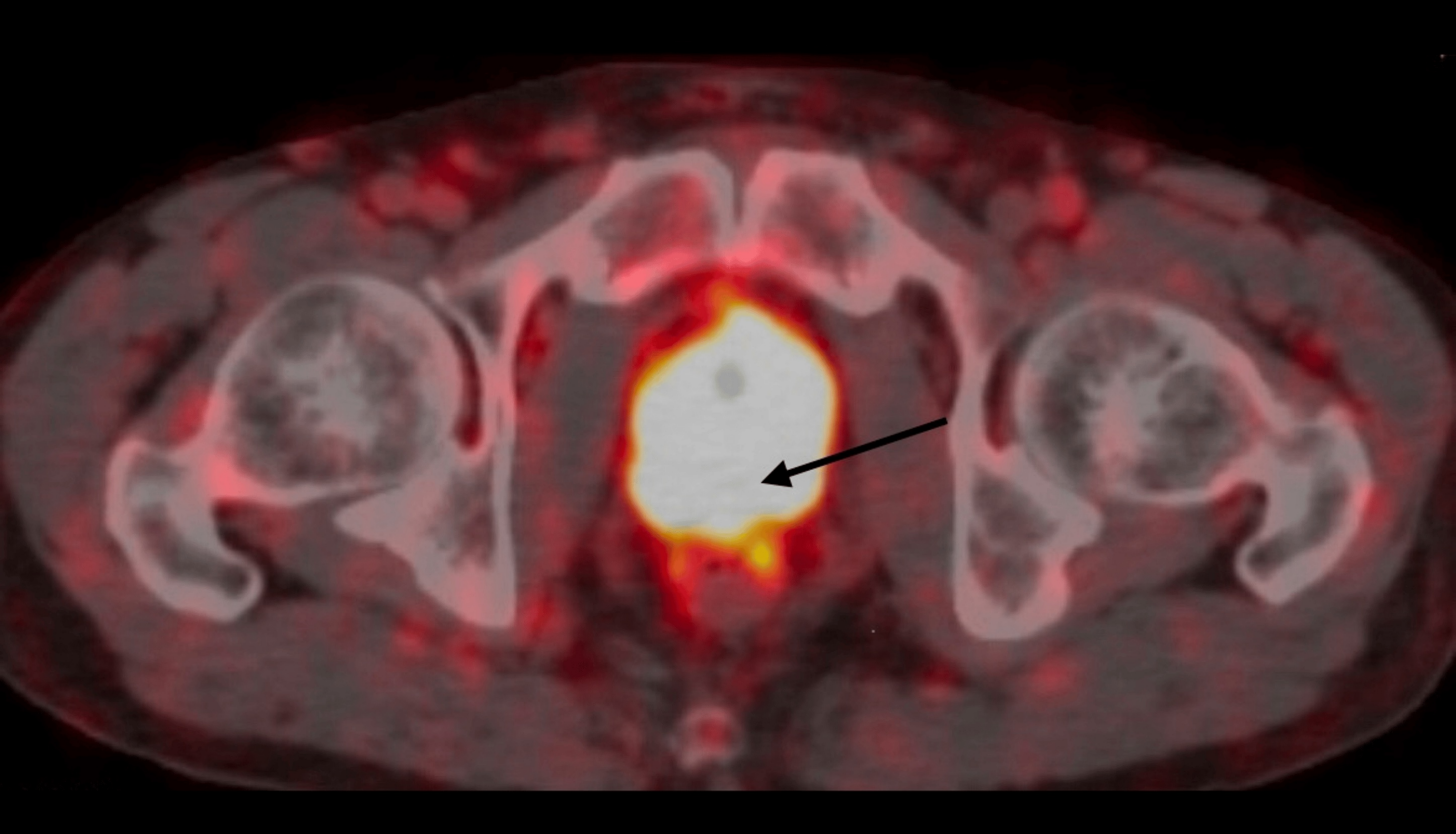
Ga-68 PSMA PET/CT revealing seminal vesicle infiltration by prostate cancer Ga-68 PSMA PET/CT imaging reveals heterogeneous tumor lesions infiltrating both seminal vesicles. The findings indicate advanced local disease with increased radiotracer uptake characteristic of prostate malignancy. CT, Computed tomography; PET, Positron emission tomography; PSMA, Prostate-specific membrane antigen

A 61-year-old male patient presented for a transrectal ultrasound-guided prostate biopsy in March 2022, prompted by an elevated prostate-specific antigen (PSA) level of 17.99 ng/mL. Pathological examination of the 12-core biopsies confirmed the presence of the International Society of Urological Pathology Grade 4 (Gleason 4+4=8) adenocarcinoma with perineural invasion (12/12 cores positive). Ga-68 prostate-specific membrane antigen (PSMA) PET/CT was planned due to suspected metastasis on bone scintigraphy. The Ga-68 PSMA PET/CT revealed heterogeneous tumor lesions (maximum standardized uptake value [SUVmax]: 19.9) infiltrating both seminal vesicles (Figure 1). Additionally, increased uptake was observed in bilateral iliac lymph nodes (SUVmax: 19.2) and a mildly elevated uptake in the middle portion of the T9 vertebral arch (SUVmax: 2.9).

**Figure 2:**
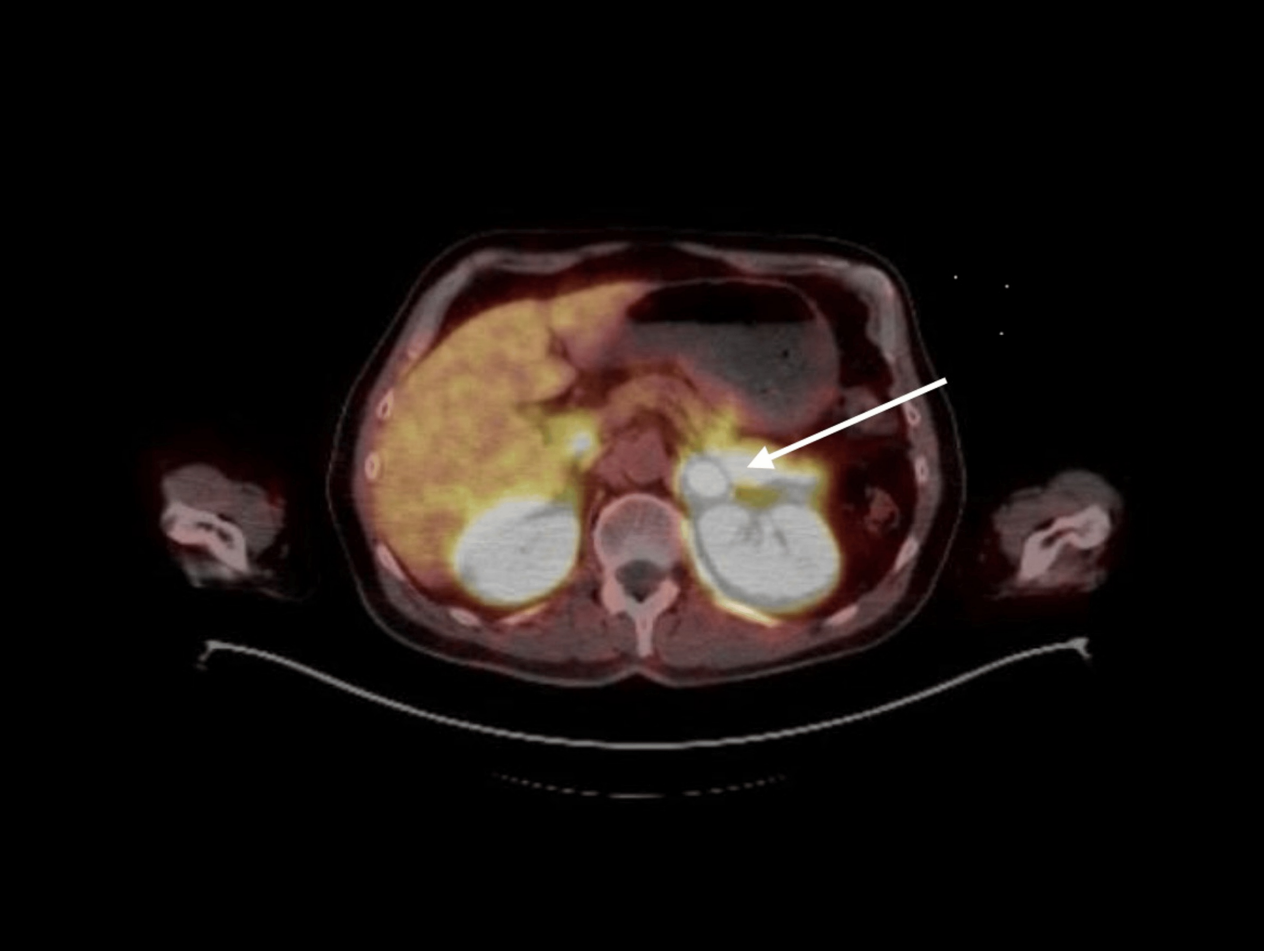
Ga-68 PSMA PET/CT image at the level of the left adrenal gland Ga-68 PSMA PET/CT imaging demonstrates nodular lesions in both adrenal glands with significantly increased radiotracer uptake. The largest lesion, located in the left adrenal gland, measures approximately 18 x 13 mm. CT, Computed tomography; PET, Positron emission tomography; PSMA, Prostate-specific membrane antigen

The treatment plan included 70 Gy of radiation to the prostate and seminal vesicles, 50.4 Gy to the pelvic lymph nodes, and 25 Gy delivered in five fractions to the T9 vertebra, administered over 28 sessions. Androgen deprivation therapy (ADT) was initiated. Following the completion of radiotherapy, the PSA level decreased to 0.1 ng/mL during subsequent follow-ups. However, in July 2023, with a rising PSA level of 2.9 ng/mL, another Ga-68 PSMA PET/CT was conducted. This imaging identified nodular lesions (SUVmax: 49.0) in both adrenal glands, the largest measuring approximately 18 × 13 mm on the left side (Figure 2). A biopsy of the left adrenal gland confirmed prostate adenocarcinoma metastasis. After consultation with endocrinology, the lesions were assessed as non-functional.

**Figure 3:**
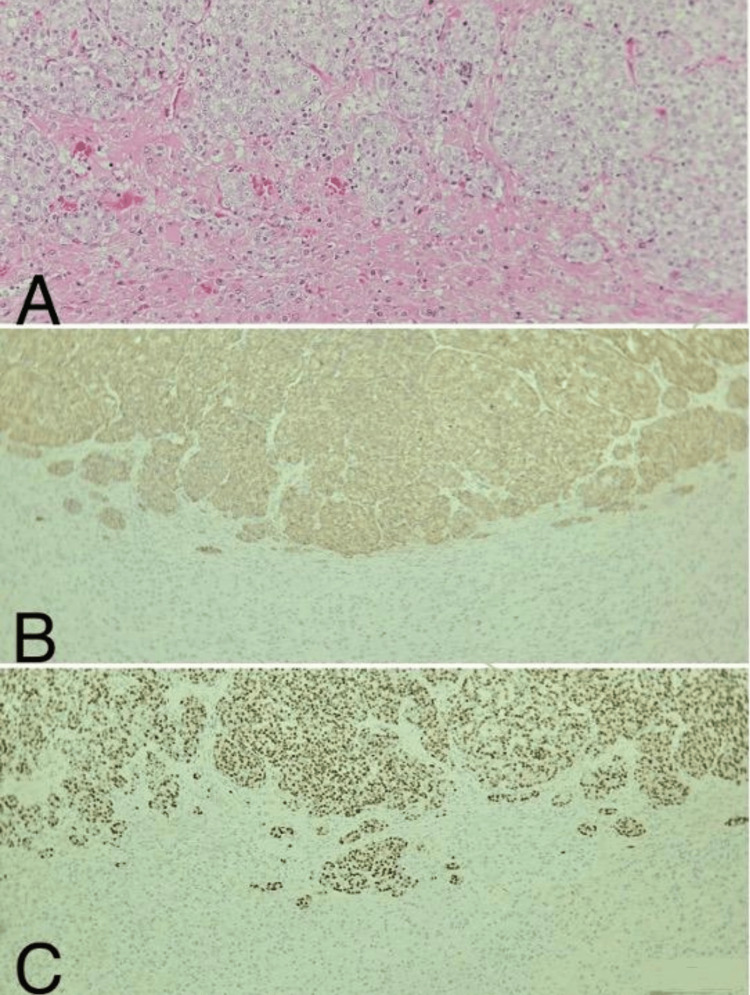
Prostate cancer metastasis in adrenal gland tissue (A) H&E, x200, (B) PSAP immunohistochemical stain, x100, (C) NKX3.1 marker, x100 Histopathological evaluation of adrenal gland tissue following bilateral adrenalectomy in September 2024 confirms metastatic prostatic adenocarcinoma. (A) Hematoxylin and eosin (H&E) staining at x200 magnification highlights the adenocarcinoma morphology. (B) Prostate-specific acid phosphatase (PSAP) immunohistochemical staining at x100 magnification demonstrates positive staining, supporting the diagnosis. (C) NK3 homeobox 1 (NKX3.1) marker staining at x100 magnification further confirms the prostatic origin of the metastatic lesion.

The patient was presented with options for surgical intervention and radiotherapy, ultimately leading to the decision for bilateral adrenalectomy. Prior to surgery, laboratory results indicated a testosterone level of 19.81 ng/dL, while the PSA level had escalated rapidly over five months to 12.77 ng/mL. Bilateral adrenalectomy was successfully performed in September 2024, with pathology confirming metastatic prostatic adenocarcinoma (Figure 3). Following surgical treatment, the patient was started on prednisolone therapy under the supervision of endocrinology physicians. One month after surgery, the PSA level decreased significantly to 0.02 ng/mL. Additionally, aldosterone levels were measured at 4.16 ng/dL, and plasma renin activity was recorded at 22.3 ng/mL/h. The patient continues to be monitored in follow-up.

## Discussion

Metastasis to the kidneys or adrenal glands is a rare occurrence, seen in approximately 1% of patients diagnosed with prostate cancer, with solitary metastases to these sites constituting only 0.3% of cases. Surgical intervention, specifically metastasectomy of adrenal metastases, has been associated with favorable biochemical responses and improved progression-free survival in patients with oligometastatic prostate cancer [3]. A study by Ost et al. demonstrated that metastasis-directed radiotherapy significantly outperformed standard follow-up in terms of ADT-free survival over a five-year period, reporting rates of 34% versus 8% (p=0.06) [4]. These findings underscore the potential benefit of aggressive management strategies for oligometastatic prostate cancer, particularly in patients presenting with isolated adrenal metastases. The role of surgical and radiotherapeutic interventions in such cases warrants further investigation, as they may offer meaningful improvements in patient prognosis and quality of life.

Isolated adrenal gland metastasis in patients with prostate cancer is exceedingly rare. Post-mortem studies reveal atypical adrenal metastases in only 2-23% of patients, typically occurring alongside bone or other distant metastases [5-7]. This highlights the unique nature of our case, in which bilateral adrenal metastasis developed in a patient with castration-resistant metastatic prostate cancer without the presence of other widespread metastatic sites.

Vinjamoori et al. analyzed 620 patients with prostate cancer and identified 85 cases of atypical metastases localized to the lungs and pleura, liver, subdiaphragmatic lymph nodes, and adrenal glands in descending order of frequency. Their study found no significant correlation between metastatic patterns and PSA levels; however, a higher Gleason score was associated with an increased likelihood of abdominal or pelvic extranodal metastasis [8].

Ga-68 PSMA uptake can demonstrate physiological activity in the adrenal glands and may also be observed in benign adrenal adenomas, occasionally leading to false-positive results [9]. Furthermore, studies have documented PSMA expression in adrenocortical carcinoma, suggesting that this type of malignancy should be considered when interpreting Ga-68 PSMA uptake in the adrenal glands [10].

In this case, a patient diagnosed with prostate cancer underwent radiotherapy and hormone therapy followed by monitoring for biochemical recurrence. Ga-68 PSMA PET/CT imaging revealed PSMA uptake in both adrenal glands. A biopsy of the adrenal gland confirmed the presence of prostate adenocarcinoma metastasis. Consequently, bilateral adrenalectomy was performed, resulting in a post-operative PSA level of 0.02 ng/mL.

## Conclusions

This case highlights the clinical rarity and therapeutic implications of isolated bilateral adrenal metastasis in castration-resistant prostate cancer. The successful management of this patient through bilateral adrenalectomy underscores the potential benefits of aggressive surgical intervention in carefully selected cases. The significant reduction in PSA levels after surgery reflects a promising biochemical response, suggesting that adrenalectomy may contribute to improved disease control and potentially enhance progression-free survival.

Given the rarity of this metastatic pattern, further research and case documentation are essential to establish standardized management protocols. This case also emphasizes the importance of multidisciplinary collaboration, including endocrinology and oncology, in tailoring treatment approaches for complex metastatic prostate cancer scenarios. These findings contribute to the limited literature on isolated adrenal metastasis and provide valuable insights into the role of metastasis-directed therapies.
